# The prognostic value of monocyte-to-lymphocyte ratio in peritoneal dialysis patients

**DOI:** 10.1186/s40001-023-01073-y

**Published:** 2023-04-10

**Authors:** Yan Yang, Yuanyuan Xu, Peiyu Lu, Hua Zhou, Min Yang, Li Xiang

**Affiliations:** Department of Nephrology, The Third Affiliated Hospital of Soochow University, No. 185 Juqian Road, Changzhou, 213003 Jiangsu China

**Keywords:** Monocyte-to-lymphocyte ratio, Mortality, Peritoneal dialysis

## Abstract

**Background:**

The monocyte-to-lymphocyte ratio (MLR) is considered as a new inflammation marker. This study was aimed to investigate the prognostic value of MLR for all-cause mortality and new-onset cardiovascular disease (CVD) events in peritoneal dialysis (PD) patients.

**Methods:**

This study enrolled patients receiving PD treatment for  ≥ 3 months. Baseline characteristics were obtained within 1 week before PD catheterization. The receiver operating characteristic curve analysis was conducted to determine the optimal cut-off value of MLR. The Kaplan–Meier curve estimated the cumulative survival rate and new CVD free survival rate. Univariate and multivariate Cox regression models were preformed to investigate the association between MLR and clinical outcomes.

**Results:**

A total of 369 PD patients participated in this study. During a median follow-up period of 32.83 months, 65 patients (24.2%) died, and 141 patients (52.4%) occurred new-onset CVD events. The Kaplan–Meier curve revealed that survival rate in high MLR group (MLR  > 0.2168) was significantly lower than in low MLR group (*P* = 0.008). Patients in high MLR group were more likely to experience CVD events (*P* = 0.002). Even after adjustment of traditional risk factors, including age, diabetes mellitus, CVD history, smoking, hyperlipidemia, high MLR remained an independent predictor of all-cause mortality [hazard ration (HR) = 2.518, 95% confidence intervals (CI) = 1.020–6.214, *P* = 0.045] and new-onset CVD events (HR = 1.815, 95% CI = 1.157–2.849, *P* = 0.010).

**Conclusions:**

This study suggested that high MLR was significantly and independently associated with all-cause mortality and CVD events in PD patients. The MLR is an inexpensive and straightforward indicator to reflect systemic inflammation status and help clinicians improve PD management.

**Supplementary Information:**

The online version contains supplementary material available at 10.1186/s40001-023-01073-y.

## Introduction

Chronic kidney disease (CKD) is an important public health problem cause of high incidence, high mortality and heavy economic and social burden. Peritoneal dialysis (PD) is a fundamental modality of renal replacement treatment for end-stage renal disease (ESRD) patients. Previous study reported the estimated systemic inflammation prevalence ranged from 12 to 65% in PD patients, depending on the different cut-off values of C-reactive protein (CRP) [1]. A variety of complex factors contribute to inflammation in PD patients, including accumulation of uremic toxins, oxidative stress, increased endotoxin level, volume overload, decreased clearance of proinflammatory cytokines, PD catheter and so on [1, 2]. Protein-energy malnutrition (PEM) and inflammation are highly prevalent and often occur concomitantly in patients with ESRD. Hence, these two conditions have been regarded together as ‘malnutrition-inflammation complex syndrome’ (MICS) [3, 4]. As is known to all, MICS is strongly correlated with higher incidence of cardiovascular disease (CVD) and mortality in dialysis patients [4–6].

Traditional inflammatory mediators, including tumor necrosis factor (TNF), CRP, and interleukin (IL) have been studied in dialysis patients. Due to high test cost and complex technological process, they have not been widely implemented in clinical practice. The monocyte-to-lymphocyte ratio (MLR), neutrophil-to-lymphocyte ratio (NLR), and platelet-to-lymphocyte ratio (PLR) are reproducible, inexpensive markers of inflammatory response. They can be easily calculated from routine blood test under simple laboratory conditions [7–9]. Recent studies have demonstrated that NLR and PLR were strongly associated with poor outcome in PD patients [8, 9]. However, few researches were focused on the prognostic value of the MLR in dialysis patients. The MLR have been confirmed as a prognostic predictor in malignancies [10, 11]. Xiang F et al. first found that higher MLR was an independent risk factor of all-cause and CVD mortality in hemodialysis (HD) patients and overwhelmed NLR [12]. Subsequently, a multicenter retrospective cohort study suggested that the highest MLR tertile was independently correlated with increased CVD mortality in PD patients [7]. At present, no studies have reported the correlation between MLR and all-cause mortality in PD patients. Therefore, this study was aimed to investigate the prognostic value of MLR for all-cause mortality and new-onset CVD events in PD patients.

## Materials and methods

### Study design and population

This was a single-center retrospective study conducted in patients initially receiving PD for at least 3 months. Adult patients (≥ 18 years old) admitted to the Department of Nephrology of the Third Affiliated Hospital of Soochow University from January, 2010 to December, 2019 were included. The exclusion criteria were as followed: (1) diagnosed with malignancies, hematological diseases or autoimmune diseases; (2) receiving corticosteroids or immunosuppressive agents within 6 months; (3) occurrence of severe infection, liver failure within 1 month; (4) lost to follow-up or transferred to other PD centers. All patients signed informed consent. The study was in keeping with the principles of the Declaration of Helsinki and with the approval of the Ethics Committee at the Third Affiliated Hospital of Soochow University, China (registration number:19/2019).

### Data collection

Baseline demographic data contained gender, age, current smoking, CVD history, comorbidities, etiology of ESRD. Clinical and biochemical data were obtained within 1 week before PD catheterization from the electronic medical record. Systolic blood pressure (SBP), diastolic blood pressure (DBP), body mass index (BMI), hemoglobin, platelet, neutrophils, lymphocytes, monocytes, albumin, globulin, serum creatine, blood urea nitrogen (BUN), uric acid, corrected calcium, phosphate, intact parathyroid hormone (iPTH), total cholesterol, triglyceride, high-density lipoprotein cholesterol (HDL-C), low-density lipoprotein cholesterol (LDL-C), apolipoprotein A1 (apo-A1), apolipoprotein B (apo-B), CRP and prealbumin were included. Baseline residual renal function (RRF) was calculated using the Chronic Kidney Disease Epidemiology Collaboration (CKD-EPI) equation [13]. MLR was calculated as monocyte count (reference range: 0.1–0.6 × 10^9^/L) divided by lymphocyte count (reference range: 1.1–3.2 × 10^9^/L).

### Clinical outcomes

All-cause mortality was the primary outcome. The secondary outcome was new-onset CVD events during follow-up. CVD events were defined as acute coronary syndrome, congestive heart failure, peripheral vascular disease, transient ischemic attack and stroke. Patients were followed-up until death, cessation of PD or the end of study period (July 31, 2021). Permanent switch to HD, renal transplantation, and recovery of renal function were considered as censored data.

### Statistical analyses

All statistical analyses were performed with SPSS 24.0 software. The optimal cut-off value of MLR was obtained from receiver operating characteristic (ROC) analysis. We performed Pearson Chi-Square test to compare categorical variables that expressed as frequencies (percentages). Normal distribution data expressed as mean ± standard deviation (SD) were compared by an unpaired t-test, while non-normal distribution data expressed as median (interquartile range, IQR) were compared by Mann–Whitney U test. We conducted the Kaplan–Meier curves and log-rank test to estimate cumulative survival rate and new CVD free survival rate. To identify the association between MLR and clinical outcomes, univariate Cox regression models were performed to calculate hazard ratio (HR) and 95% confidence intervals (CI). Then, multivariate Cox regression model with Forward Stepwise (Conditional) was used to examine whether MLR was an independent risk factor. The variables with *P* < 0.1 by univariate analysis and recognized prognostic indicators were included. A two-sided *P*-value less than 0.05 indicated statistically significant difference.

## Results

### Baseline characteristics of the study population

A total of 369 incident PD patients were enrolled in this study. The median age was 47 (37, 59) years, 58.8% of patients were male. Sixty-one patients (16.5%) complicated with diabetes mellitus and 41 patients (11.1%) had CVD history. ROC curve analysis indicated the optimal cut-off value was 0.2168 for MLR. Patients were separated into high MLR group (> 0.2168) and low MLR group (≤ 0.2168). There were 270 patients in high MLR group and 99 patients in low MLR group.

The baseline characteristics are listed in Table 1. There was no significant difference in demographic characteristics between two groups, except for age. Patients in high MLR group were older than those in low MLR group (*P* = 0.003). With respect to routine blood test, the high MLR group displayed significantly higher white blood count (*P* = 0.003), higher neutrophil count (*P* < 0.001), lower lymphocyte count (*P* < 0.001), and higher monocyte count (*P* < 0.001). Compared with low MLR group, patients with high MLR showed lower serum albumin (*P* < 0.001) and lower corrected calcium (*P* = 0.005). No significant differences were detected in ESRD causes and other laboratory data.

**Table 1:** Demographic characteristics and laboratory data of the study population

Clinical characteristics	Low MLR group (n = 99)	High MLR group (*n* = 270)	*P*-value
Male (*n*, %)	50 (50.5%)	164 (60.7%)	0.078
Age (years)	41 (34, 54)	49 (39, 61)	0.003
BMI (kg/m^2^)	22.18 ± 2.85	22.62 ± 3.04	0.214
SBP (mmHg)	153 (139, 164)	156 (140, 170)	0.061
DBP (mmHg)	90 (81, 97)	89 (80, 100)	0.555
Diabetes mellitus (n, %)	13 (13.1%)	48 (17.8%)	0.287
Current smoking (n, %)	11 (11.1%)	45 (16.7%)	0.188
CVD history (n, %)	9 (9.1%)	32 (11.9%)	0.455
ESRD causes			0.699
Glomerulonephritis (n, %)	56 (56.6%)	149 (55.2%)	
Diabetic nephropathy (n, %)	7 (7.1%)	27 (10.0%)	
Hypertension nephropathy (n, %)	12 (12.1%)	24 (8.9%)	
Other/unknown (n, %)	24 (24.2%)	70 (25.9%)	
White blood count (× 10^9^/L)	5.59 (4.60, 6.97)	6.25 (5.09, 8.01)	0.003
Hemoglobin (g/dL)	8.20 (7.00, 9.40)	8.15 (6.80, 9.30)	0.431
Platelet count (× 10^9^/L)	160 (122, 211)	161 (126, 207)	0.701
Neutrophil count (× 10^9^/L)	3.38 (2.94, 4.91)	4.45 (3.45, 5.86)	< 0.001
Lymphocyte count (× 10^9^/L)	1.44 (1.09, 1.87)	1.05 (0.74, 1.39)	< 0.001
Monocyte count (× 10^9^/L)	0.25 (0.18, 0.31)	0.40 (0.31, 0.52)	< 0.001
Albumin (g/L)	35.6 (32.9, 38.3)	32.3 (28.9, 35.6)	< 0.001
Globulin (g/L)	25.5 (22.3, 29.9)	25.9 (22.9, 29.0)	0.971
BUN (mmol/L)	29.81 ± 10.67	30.75 ± 10.38	0.444
Serum creatinine (μmol/L)	765.0 (622.4, 931.0)	803.5 (685.5, 1000.5)	0.125
Uric acid (μmol/L)	484.38 ± 130.50	50.28 ± 136.34	0.316
RRF (ml/min/1.73m^2^)	5.72 (4.59, 7.14)	5.30 (4.23, 6.95)	0.127
Corrected calcium (mmol/L)	2.11 (1.83, 2.25)	1.99 (1.78, 2.16)	0.005
Phosphate (mmol/L)	1.88 (1.47, 2.32)	1.90 (1.58, 2.37)	0.311
iPTH (Pg/mL)	254.3 (131.4, 420.6)	233.8 (92.6, 418.4)	0.239
Prealbumin (mg/L)	269 (224, 313)	269 (220, 303)	0.615
CRP (mg/L)	4.8 (3.3, 7.0)	5.0 (3.7, 9.6)	0.110
Cholesterol (mmol/L)	4.00 (3.43, 4.92)	4.18 (3.50, 4.85)	0.451
Triglyceride (mmol/L)	1.56 (1.05, 2.11)	1.78 (1.22, 2.41)	0.058
HDL-C (mmol/L)	0.94 (0.76, 1.16)	0.92 (0.76, 1.11)	0.748
LDL-C (mmol/L)	2.03 (1.61, 2.57)	2.08 (1.68, 2.47)	0.875
apo-A1 (g/L)	1.11 ± 0.23	1.14 ± 0.19	0.169
apo-B (g/L)	0.81 (0.67, 1.00)	0.88 (0.73, 1.01)	0.092

### MLR and all-cause mortality

**Fig. 1:**
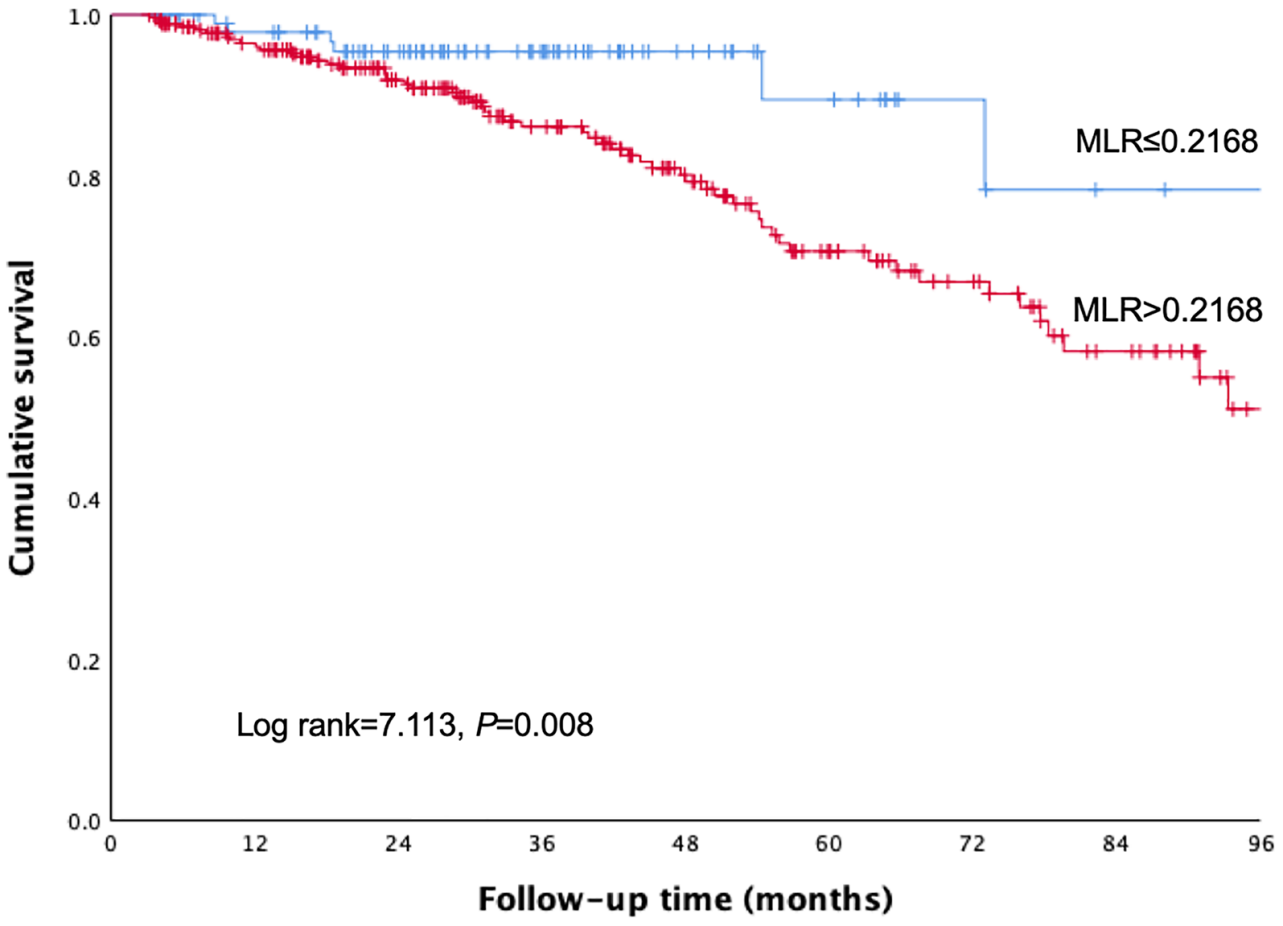
Kaplan–Meier curves for all-cause mortality in patients undergoing PD according to MLR

At the end of this study, 65 patients (24.2%) died, 119 patients (44.2%) transferred to HD, 41 patients (15.2%) received renal transplantation and 1 patient (0.4%) recovered normal renal function. The cumulative survival rates at 1, 3, and 5 years were 97.8%, 95.4%, and 89.5% in low MLR group; 96.5%, 86.1%, and 70.5% in high MLR group, respectively. The Kaplan–Meier curve revealed significant different survival rate between MLR groups (Fig. 1, Log rank = 7.113, *P* = 0.008).

Table 2 indicates the association between baseline characteristics and all-cause mortality. We detected that age over 60 years old (HR = 4.487, 95% CI = 2.728–7.379, *P* < 0.001), complicated with diabetes mellitus (HR = 3.687, 95% CI = 2.146–6.335, *P* < 0.001), current smoking status (HR = 2.132, 95% CI = 1.195–3.804, *P* = 0.010), history of CVD (HR = 3.033, 95% CI = 1.738–5.292, *P* < 0.001), hemoglobin (HR = 1.040, 95% CI = 1.007–1.075, *P* = 0.018), platelet (HR = 1.006, 95% CI = 1.003–1.010, *P* = 0.001), MLR (HR = 2.983, 95% CI = 1.284–6.931, *P* = 0.011), globulin (HR = 1.056, 95% CI = 1.007–1.106, *P* = 0.024), CRP (HR = 1.021, 95% CI = 1.008–1.034, *P* = 0.002), cholesterol (HR = 1.274, 95% CI = 1.035–1.567, *P* = 0.022), LDL-C (HR = 1.555, 95% CI = 1.156–2.092, *P* = 0.004), and apo-B (HR = 3.840, 95% CI = 1.546–9.541, *P* = 0.004) were associated with increased mortality risk. Serum creatinine (HR = 0.999, 95% CI = 0.998–1.000, *P* = 0.035) and prealbumin (HR = 0.992, 95% CI = 0.988–0.996, *P* < 0.001) were negatively associated with all-cause mortality.

**Table 2:** Univariate Cox analysis of clinical outcomes in peritoneal dialysis patients

Variables	All-cause mortality		new-onset CVD events	
	Univariate analysis		Univariate analysis	
	HR (95% CI)	*P-*value	HR (95% CI)	*P-*value
Gender (male)	0.773 (0.471, 1.268)	0.308	0.777 (0.554, 1.090)	0.144
Age (≥ 60 years old)	4.487 (2.728, 7.379)	< 0.001	2.514 (1.798, 3.515)	< 0.001
BMI (kg/m^2^)	1.078 (0.995, 1.167)	0.065	1.095 (1.037, 1.157)	0.001
SBP (mmHg)	1.006 (0.995, 1.016)	0.301	1.010 (1.003, 1.017)	0.008
DBP (mmHg)	0.989 (0.973, 1.006)	0.199	0.993 (0.982, 1.005)	0.261
Diabetes mellitus	3.687 (2.146, 6.335)	< 0.001	3.437 (2.348, 5.031)	< 0.001
Current smoking	2.132 (1.195, 3.804)	0.010	1.605 (1.046, 2.462)	0.030
CVD history	3.033 (1.738, 5.292)	< 0.001	2.461 (1.615, 3.748)	< 0.001
White blood count (× 10^9^/L)	1.064 (0.961, 1.178)	0.230	1.090 (1.013, 1.173)	0.022
Hemoglobin (g/dL)	1.040 (1.007, 1.075)	0.018	1.028 (1.001, 1.056)	0.045
Platelet count (× 10^9^/L)	1.006 (1.003, 1.010)	0.001	1.004 (1.001, 1.007)	0.003
Neutrophil count (× 10^9^/L)	1.081 (0.975, 1.198)	0.138	1.088 (1.006, 1.177)	0.034
Lymphocyte count (× 10^9^/L)	0.736 (0.435, 1.245)	0.253	1.065 (0.766, 1.479)	0.709
Monocyte count (× 10^9^/L)	2.095 (0.623, 7.047)	0.232	2.265 (1.066, 4.811)	0.033
MLR (> 0.2168)	2.983 (1.284, 6.931)	0.011	2.010 (1.285, 3.146)	0.002
Albumin (g/L)	0.976 (0.929, 1.026)	0.338	0.954 (0.922, 0.986)	0.006
Globulin (g/L)	1.056 (1.007, 1.106)	0.024	1.035 (1.000, 1.071)	0.050
BUN (mmol/L)	0.978 (0.953, 1.004)	0.101	0.992 (0.975, 1.008)	0.315
Serum creatinine (μmol/L)	0.999 (0.998, 1.000)	0.035	1.000 (0.999, 1.000)	0.616
Uric acid (μmol/L)	0.999 (0.997, 1.001)	0.161	1.000 (0.999, 1.002)	0.510
RRF (ml/min/1.73m^2^)	1.051 (0.933, 1.183)	0.413	0.977 (0.898, 1.063)	0.587
Corrected calcium (mmol/L)	1.047 (0.507, 2.161)	0.902	0.854 (0.534, 1.367)	0.510
Phosphate (mmol/L)	0.892 (0.597, 1.333)	0.577	0.998 (0.761, 1.308)	0.986
iPTH (Pg/mL)	1.000 (0.999, 1.001)	0.537	1.000 (0.999, 1.000)	0.182
Prealbumin (mg/L)	0.992 (0.988, 0.996)	< 0.001	0.997 (0.994, 1.000)	0.030
CRP (mg/L)	1.021 (1.008, 1.034)	0.002	1.012 (1.001, 1.022)	0.027
Cholesterol (mmol/L)	1.274 (1.035, 1.567)	0.022	1.305 (1.138, 1.497)	< 0.001
Triglyceride (mmol/L)	1.009 (0.806, 1.263)	0.939	1.229 (1.089, 1.389)	0.001
HDL-C (mmol/L)	1.439 (0.659, 3.144)	0.362	0.691 (0.382, 1.250)	0.221
LDL-C (mmol/L)	1.555 (1.156, 2.092)	0.004	1.279 (1.047, 1.563)	0.016
apo-A1 (g/L)	1.219 (0.309, 4.804)	0.777	1.885 (0.812, 4.378)	0.140
apo-B (g/L)	3.840 (1.546, 9.541)	0.004	2.877 (1.608, 5.149)	< 0.001

After adjustment for these cofounding factors, multivariate Cox regression model revealed that high MLR (HR = 2.518, 95% CI = 1.020–6.214, *P* = 0.045), age (HR = 3.791, 95% CI = 2.261–6.357, *P* < 0.001), diabetes (HR = 1.884, 95% CI = 1.049–3.382, *P* = 0.034), CVD history (HR = 2.374, 95% CI = 1.326–4.249, *P* = 0.004), hemoglobin (HR = 1.058, 95% CI = 1.018–1.099, *P* = 0.004), CRP (HR = 1.016, 95% CI = 1.002–1.030, *P* = 0.021), and LDL-C (HR = 1.620, 95% CI = 1.157–2.269, *P* = 0.005) were independently and significantly related to all-cause mortality (Table 3). ROC curves indicated the area under curve for MLR was larger than CRP (Additional file 1: Fig. S1, 0.615 vs. 0.599).

**Table 3:** Multivariate Cox analysis of all-cause mortality in peritoneal dialysis patients

Variables	Multivariate analysis	
	HR (95% CI)	*P-*value
Age (≥ 60 years old)	3.791 (2.261, 6.357)	< 0.001
Diabetes mellitus	1.884 (1.049, 3.382)	0.034
CVD history	2.374 (1.326, 4.249)	0.004
Hemoglobin (g/dL)	1.058 (1.018, 1.099)	0.004
MLR (> 0.2168)	2.518 (1.020, 6.214)	0.045
CRP (mg/L)	1.016 (1.002, 1.030)	0.021
LDL-C (mmol/L)	1.620 (1.157, 2.269)	0.005

### MLR and new-onset CVD events

**Fig. 2:**
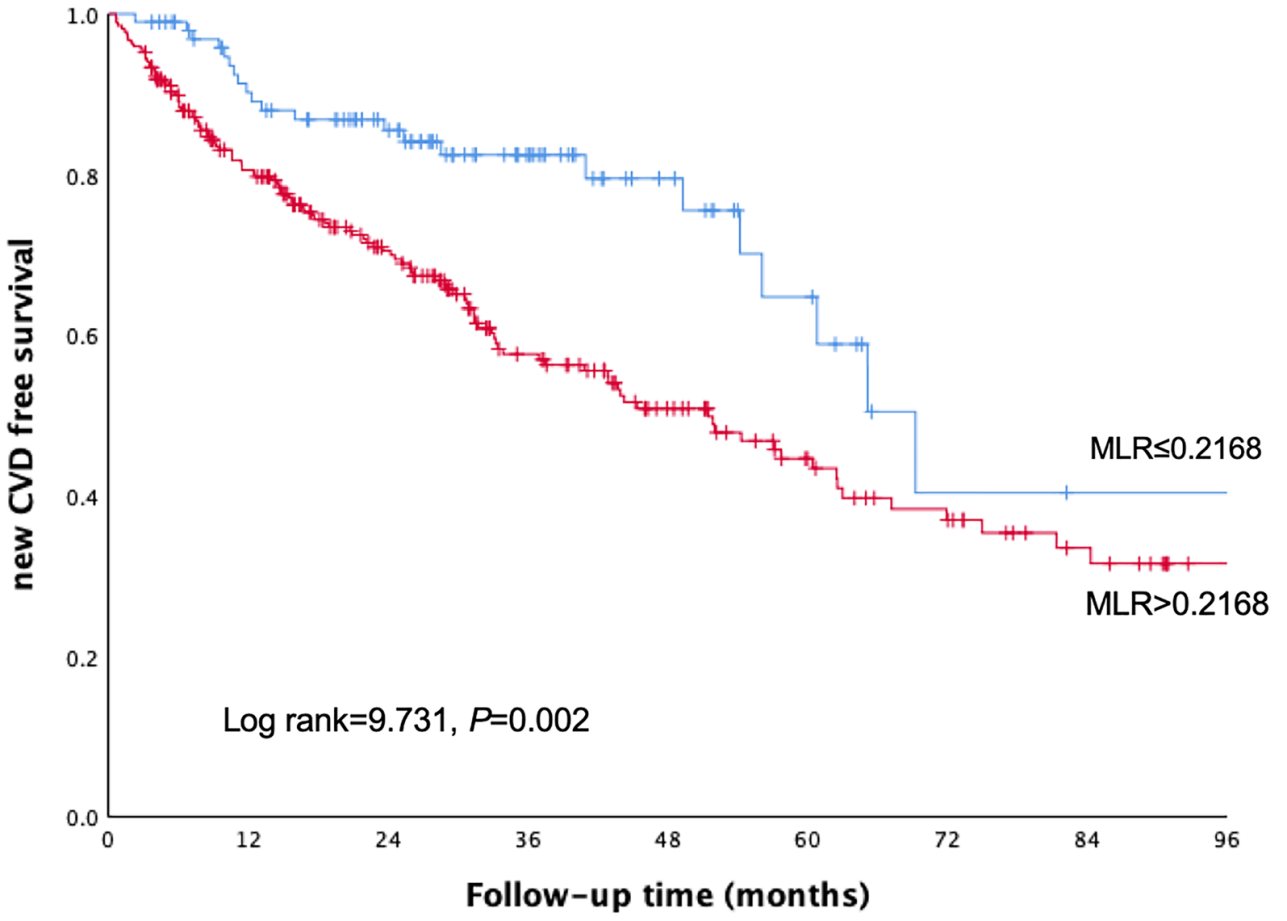
Kaplan–Meier curves for new-onset CVD events in patients undergoing PD according to MLR

During a median follow-up period of 32.83 months, 141 patients (52.4%) occurred new-onset CVD events. Among 141 CVD events, 23 (16.3%) were happened in low MLR group, and 118 (83.7%) were happened in high MLR group. The Kaplan–Meier curve comparing CVD events is shown in Fig. 2. Patients in high MLR group were more likely to experience CVD events (43.7% vs. 23.2%, Log rank = 9.731, *P* = 0.002).

The results of univariate Cox regression models to investigate risk factor of CVD events are shown in Table 2. There were 19 variables associated with the incidence of CVD, including age (HR = 2.514, 95% CI = 1.798–3.515, *P* < 0.001), BMI (HR = 1.095, 95% CI = 1.037–1.157, *P* = 0.001), SBP (HR = 1.010, 95% CI = 1.003–1.017, *P* = 0.008), diabetes mellitus (HR = 3.437, 95% CI = 2.348–5.031, *P* < 0.001), current smoking status (HR = 1.605, 95% CI = 1.046–2.462, *P* = 0.030), CVD history (HR = 2.461, 95% CI = 1.615–3.748, *P* < 0.001), white blood count (HR = 1.090, 95% CI = 1.013–1.173, *P* = 0.022), hemoglobin (HR = 1.028, 95% CI = 1.001–1.056, *P* = 0.045), platelet (HR = 1.004, 95% CI = 1.001–1.007, *P* = 0.003), neutrophil (HR = 1.088, 95% CI = 1.006–1.177, *P* = 0.034), monocyte (HR = 2.265, 95% CI = 1.066–4.811, *P* = 0.033), MLR (HR = 2.010, 95% CI = 1.285–3.146, *P* = 0.002), albumin (HR = 0.954, 95% CI = 0.922–0.986, *P* = 0.006), prealbumin (HR = 0.997, 95% CI = 0.994–1.000, *P* = 0.030), CRP (HR = 1.012, 95% CI = 1.001–1.022, *P* = 0.027), cholesterol (HR = 1.305, 95% CI = 1.138–1.497, *P* < 0.001), triglyceride (HR = 1.229, 95% CI = 1.089–1.389, *P* = 0.001), LDL-C (HR = 1.279, 95% CI = 1.047–1.563, *P* = 0.016), and apo-B (HR = 2.877, 95% CI = 1.608–5.149, *P* < 0.001).

Furthermore, multivariate Cox regression model analysis detected high MLR (HR = 1.815, 95% CI = 1.157–2.849, *P* = 0.010) was an independent predictor of new-onset CVD events, along with age (HR = 2.172, 95% CI = 1.511–3.123, *P* < 0.001), BMI (HR = 1.061, 95% CI = 1.000–1.126, *P* = 0.049), diabetes (HR = 1.847, 95% CI = 1.194–2.858, *P* = 0.006), CVD history (HR = 2.163, 95% CI = 1.382–3.386, *P* = 0.001), SBP (HR = 1.009, 95% CI = 1.001–1.016, *P* = 0.030), and cholesterol (HR = 1.325, 95% CI = 1.146–1.531, *P* < 0.001) (Table 4).

**Table 4:** Multivariate Cox analysis of new-onset CVD events in peritoneal dialysis patients

Variables	Multivariate analysis	
	HR (95% CI)	*P-*value
Age (≥ 60 years old)	2.172 (1.511, 3.123)	< 0.001
BMI (kg/m^2^)	1.061 (1.000, 1.126)	0.049
Diabetes mellitus	1.847 (1.194, 2.858)	0.006
CVD history	2.163 (1.382, 3.386)	0.001
SBP (mmHg)	1.009 (1.001, 1.016)	0.030
MLR (> 0.2168)	1.815 (1.157, 2.849)	0.010
Cholesterol (mmol/L)	1.325 (1.146, 1.531)	< 0.001

## Discussion

The main finding of this study was that baseline high MLR level (> 0.2168) was an independent predictor for all-cause mortality and CVD events, even after adjustment of traditional risk factors, including age, diabetes mellitus, CVD history, smoking, hyperlipidemia.

The PD favored policy in China has made contribution to increased utilization of PD. In recent decades, there was a significant reduction of mortality risk in PD patients [14]. Nowadays, most studies reported similar survival rate in PD and in-center HD [14–16]. Despite all this, the mortality of PD is still higher than age-matched general population [2]. In our study, the 5-year survival rate was lower in high MLR group than in low MLR group (70.5% vs. 89.5%). We first demonstrated that high MLR was significantly and independently associated with a HR for all-cause mortality of 2.518 (95%CI = 1.020–6.214, *P* = 0.045) after adjusting recognized risk factors. It is worth noting that inflammation plays an important role in the pathogenesis and progression of CKD [17]. Enhancement of inflammation or oxidative stress, generation of proinflammatory cytokines and immune system change can result in nephron loss and kidney injury [18]. Naicker SD et al. compared leucocyte and monocyte populations in patients with CKD stage 1–5 and healthy controls [19]. They confirmed dysregulation of neutrophil and monocyte subset in CKD and found a distinct subpopulation of intermediate monocytes was significantly associated with estimated glomerular filtration rate (eGFR) [19]. A large longitudinal cohort study enrolled 11,280 participants and detected increased MLR was associated with new-onset CKD [20]. The predictive value of MLR for all-cause mortality has been substantiated in HD patients [12]. Our results suggested this conclusion could be applicable to PD patients.

CVD is a severe complication and leading cause of death, occupying nearly 60% of all-cause mortality in PD [7]. The baseline MLR has been found to predict CVD mortality in dialysis patients in previous studies [7, 12]. A retrospective study with 543 patients undergoing coronary angiography observed that MLR was an independent risk factor of coronary artery disease and had better predictive value of lesion severity than NLR [21]. Increased lymphocytes apoptosis causes lymphocytopenia that can reflect a poorly regulated immune response and impaired coronary microcirculation [22]. Monocytes involve in the formation and rupture of atherosclerotic plaque by recruiting to the artery wall, differentiating into macrophages and activating the production of matrix metalloproteinases, pro-inflammatory cytokines secretion, and reactive oxidative species [23]. Both the components of MLR were confirmed to be independent predictors of CVD [24]. Therefore, we speculate that MLR can be recognized as a better indicator than monocytes or lymphocytes separately. In our PD center, more than half of the patients occurred CVD and high MLR was associated with increased incidence of CVD (HR = 1.815, 95%CI = 1.157–2.849, *P* = 0.010).

Consistent with previous study, elevated CRP was found to predict all-cause death in PD [25]. We detected every 1 mg/L increase in CRP was independently related to higher all-cause mortality (HR = 1.016, 95%CI = 1.002–1.030, *P* = 0.021). In addition, the MLR was superior to CRP in predicting mortality according to ROC curves. We also detected increased CRP was associated with CVD, but no statistical significance was found in multivariate Cox analysis. Taken together, our study provided convinced evidences that MLR as a novel marker had a better kinetic pattern than CRP.

Anemia is not only a common complication of CKD, but also a risk factor for CVD and mortality [26]. It was an interesting finding that hemoglobin exhibited an incremental association with the mortality and CVD risk in univariate Cox analysis. Noteworthily, we only investigated the hemoglobin level before PD, further studies should integrate the treatment of hemoglobin and its variability. Dysregulation of lipid metabolism participates in CKD-associated inflammation and oxidative stress which promote CVD [27]. Experimental study demonstrated that reduced cholesterol and LDL-C could limit CVD and mortality in the general population [28]. Our study revealed that increased LDL-C was independently related to all-cause mortality (HR = 1.620, 95%CI = 1.157–2.269, *P* = 0.005), while elevated cholesterol was an independent risk factor for CVD (HR = 1.325, 95%CI = 1.146–1.531, *P* < 0.001). A prospective cohort study with 1616 PD patients showed that the highest LDL-C to HDL-C ratio tertile was significantly associated with all-cause and CVD mortality [29]. Another study analyzed data from Taiwan Renal Registry Data System and indicated that risk of death rose with a higher cholesterol level [30]. Stepanova N et al. conducted a cross-sectional pilot study and first demonstrated there was really a correlation between dyslipidemia and intraperitoneal inflammation in PD patients [31]. It seemed that lipid profiles had prognostic value in risk prediction. However, lipid-lowering therapy should be considered to determine the significance of dyslipidemia in future researches.

There are still several limitations in this study. First, because this is a single-center retrospective study conducted in China with a relatively small sample, its potential selection bias and center-specific effect may limit external validity and statistical power. A second issue is lack of comparison with traditional inflammation biomarkers, such as IL-6, TNF-α. Although prognostic values of them have been proved in numerous studies [2, 32], they are not measured routinely because of expensive and inconvenient assays. Besides, the optimal cut-off value of the MLR needs further validation in other ethnic cohorts. Because of competing risk such as HD transfer, kidney transplantation, the incidence of all-cause mortality and CVD risk may potentially be inflated by Kaplan–Meier method. Finally, we only investigated the effects of baseline variables, the influences of longitudinal changes have been ignored. Future studies should pay more attention to time-varying MLR and determine the causal relationship between MLR and clinical outcomes in PD patients.

## Conclusions

This study first demonstrated that high MLR was significantly and independently correlated with all-cause mortality and CVD events in PD patients despite adjustments for multiple confounders. We suggested a possible potential predictive value for MLR was superior to monocytes, lymphocytes, and CRP. The MLR is an inexpensive and straightforward indicator to reflect systemic inflammation status. Future researches integrating baseline and changes over time are needed to identify the clinical significance of MLR and help clinicians improve PD management.

## Supplementary Information

**Additional file 1: Figure S1.** ROC curves of the probability of MLR and CRP in predicting all-cause mortality.

## Data Availability

The data of this study are available from the corresponding author upon request.
