# Supplementary figures and images for: The prognostic value of monocyte-to-lymphocyte ratio in peritoneal dialysis patients
Source: Eur J Med Res. 2023 Apr 10;28:152. doi: 10.1186/s40001-023-01073-y (PMC10084613; doi:10.1186/s40001-023-01073-y)

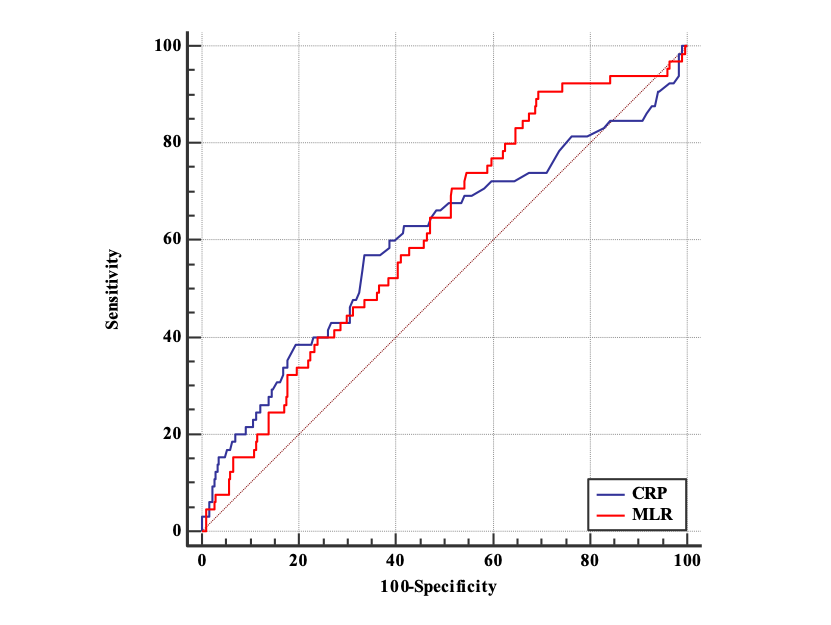

Supplement: Supplementary file 1 — Additional file 1: Figure S1. ROC curves of the probability of MLR and CRP in predicting all-cause mortality. [file 40001_2023_1073_MOESM1_ESM.png]
